# The Polycomb Repressive Complex 2 Is a Potential Target of SUMO Modifications

**DOI:** 10.1371/journal.pone.0002704

**Published:** 2008-07-16

**Authors:** Eva Madi Riising, Roberto Boggio, Susanna Chiocca, Kristian Helin, Diego Pasini

**Affiliations:** 1 Biotech Research and Innovation Centre (BRIC), University of Copenhagen, Copenhagen, Denmark; 2 Centre for Epigenetics, University of Copenhagen, Copenhagen, Denmark; 3 Department of Experimental Oncology, European Institute of Oncology, Milan, Italy; University of Munich and Center of Integrated Protein Science, Germany

## Abstract

**Background:**

The Polycomb Repressive Complex 2 (PRC2) functions as a transcriptional repressor through a mechanism that involves methylation of Histone H3 at lysine 27. The PRC2 complex activity is essential for cellular proliferation, development, and cell fate decisions. PRC2 target genes include important regulators of development and proliferation as well as tumor suppressor genes. Consistent with this, the activity of several Polycomb group (PcG) proteins is deregulated in human cancer suggesting an important role for PcGs in tumor development. Whereas the downstream functions of PcGs are well characterized, the mechanisms of their recruitment to target genes and the regulation of their activity are not fully understood.

**Principal Findings:**

Here we show that the two PRC2 components SUZ12 and EZH2 are sumoylated *in vitro* and *in vivo*. Among several putative sumoylation sites we have mapped the major site of SUZ12 sumoylation. Furthermore, we show that SUZ12 interacts with the E2-conjugating enzyme UBC9 both *in vitro* and *in vivo* and that mutation of the SUZ12 sumoylation site does not abolish this binding. Finally, we provide evidence that the E3-ligase PIASXβ interacts and enhances the sumoylation of SUZ12 *in vivo* suggesting that PIASXβ could function as an E3-ligase for SUZ12.

**Conclusions:**

Taken together, our data identify sumoylation as a novel post-translational modification of components of the PRC2 complex, which could suggest a potential new mechanism to modulate PRC2 repressive activity. Further work aimed to identify the physiological conditions for these modifications will be required to understand the role of SUZ12 and EZH2 sumoylation in PcG-mediated epigenetic regulation of transcription.

## Introduction

Polycomb group proteins (PcG) are evolutionarily conserved regulators of development [1]–[5]. PcGs function as transcriptional repressors and directly regulate the expression of genes involved in differentiation, development, cell fate decisions and stem cell self renewal [6]–[8].

PcGs form two distinct multiprotein complexes named–Polycomb Repressive Complex 1 and 2 (PRC1 and PRC2). PRC1 is a large complex and consists of more than 10 different subunits including the oncoprotein BMI1, CBX2, CBX4, CBX7, CBX8, SCML, HPH1-3 and RING1A-B [9], [10]. The PRC1 complex catalyzes the ubiquitylation of histone H2A through the ubiquitin E3 ligase activity of the RING1A and RING1B subunits [11] which may lead to gene silencing through the induction of chromatin compaction [12].

PRC2 is a smaller and highly conserved complex. The core of the PRC2 complex is formed by the three PcG proteins EZH2, EED and SUZ12 and by the histone binding protein RbAp48/46 [13]–[16]. The PRC2 complex catalyzes the tri-methylation (me3) of histone H3 on Lysine (K) 27 [13]–[16]. The activity of PRC2 is required for the recruitment of PRC1 to target genes [11] through a mechanism that most likely involves the binding of PRC1 to H3K27me3 [15], [16]. Although EZH2 is the catalytic component of PRC2, all three PcG components of the PRC2 complex are essential for EZH2 Lysine histone Methyl Transferase (KMT) activity and for mouse embryonic development [3], [17].

The expression of *EZH2*, *EED,* and *SUZ12* is controlled by the pRB/E2F pathway and PRC2 activity is essential for the proliferation of primary and cancer cell lines [3], [18]. Consistent with this, direct deregulation of different PcGs have been reported in human cancers [3], [19]. We and others have demonstrated that BMI1, CBX7, CBX8 and EZH2 have growth promoting and oncogenic effects, which in part can be ascribed to the ability of PcGs to repress the expression of the tumor suppressor proteins p16 and ARF [9], [18], [20], [21].

Despite the key role of the PcGs in regulating cellular homeostasis, we know relatively little about the mechanisms by which PcG-mediated silencing is established and maintained. Recent studies have shown that AKT-dependent EZH2 phosphorylation inhbits EZH2 recruitment to chromatin and thereby indirectly its repressive activity [22]. It is likely that other post-translational modifications could be involved in the regulation of PcG activity. For example, it was shown that CBX4/HPC2, a component of the PRC1 complex, is sumoylated and functions as a SUMO E3 ligase facilitating the SUMO modification of the transcriptional repressor CtBP and of the kinase HIPK2 [23], [24].

The conjugation of SUMO to target proteins involves four enzymatic steps after which SUMO is covalently attached by an isopeptidic bond to a lysine residue [25]–[27]. The target lysine is often found in a consensus sequence ΨKXE/D. A recent study has revealed an extended consensus motif consisting of a cluster of acidic residues downstream of the ΨKXE/D motif leading to a more accurate prediction of SUMO modification sites [28]. An ATP dependent cascade of the E1 activating enzyme (SAE1-SAE2), the E2 conjugating enzyme (UBC9) and of E3 ligases, covalently binds the C-terminal cystein of the small SUMO protein to the target lysine [26]. Protein modification by sumoylation can have several different outcomes. Reported effects of sumoylation include change of conformation, stability, interactions and localization of the target proteins [25]–[27], [29]. Several transcription factors have been reported to be SUMO modified and such modifications mainly correlate with repression of transcription [27], [29].

The identification of novel post-translational modifications of PcG proteins could improve our understanding of the mechanisms of PcG-mediated epigenetic regulation of transcription. Here we demonstrate that two subunits of the PRC2 complex (SUZ12 and EZH2) are sumoylated *in vitro* and *in vivo*. Moreover, we map the site of sumoylation in SUZ12 and we show that SUZ12 can interact with UBC9 *in vivo* and *in vitro*. Furthermore, we have investigated the upstream regulatory pathways to SUZ12 sumoylation and found that the E3 ligase PIASXβ, but not CBX4, enhances the sumoylation of SUZ12. Together, our data identifies sumoylation as a novel post-translational modification of different PRC2 components, which could open new possibilities to understand the regulation of PcG activities.

## Results

### EZH2 and SUZ12 are sumoylated *in vivo* and *in vitro*

**Figure 1:**
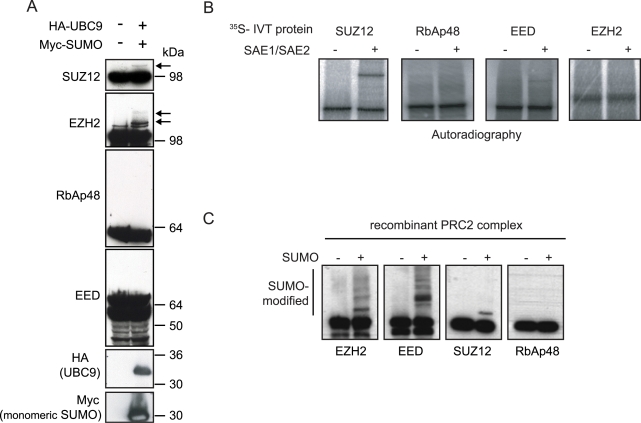
SUZ12 and EZH2 are sumoylated *in vivo* and *in vitro.* (A) Western blot analysis of 293T cells transfected with SUMO-1 and UBC9 showing sumoylation of endogenous SUZ12 and EZH2. Arrows indicate the SUMO modified forms. Molecular weight markers are indicated to the right. (B) *In vitro* sumoylation assay of ^35^S-labeled *in vitro* translated SUZ12, RbAp48, EED and EZH2, showing sumoylation of SUZ12 (C) *In vitro* sumoylation assay of the recombinant PRC2 complex showing sumoylation of SUZ12, EZH2 and EED.

To understand if members of the PRC2 complex are potential targets for sumoylation, we analyzed the amino acid sequence of SUZ12, EZH2, EED and RbAp48 with the SUMOplot™Analysis Program (Abgent; www.abgent.com/doc/sumoplot). This analysis revealed that all four proteins contain one or more SUMO consensus motifs (data not shown), suggesting that all components of the PRC2 complex could be targets of sumoylation. Thus, we tested if endogenous SUZ12, EZH2, EED and RbAp48 could be SUMO modified *in vivo*. Most of the sumoylated proteins reported in the literature present a very low pool of protein conjugated with SUMO [25]. In order to raise the proportion of modified endogenous substrate we transfected 293T cells with SUMO-1 and UBC9 (Fig. 1A). Western blot analysis detected slower migrating forms of SUZ12 and EZH2 upon SUMO and UBC9 expression with a molecular weight consistent with SUMO-conjugation (Fig. 1A). EED is present in the cells in at least 4–5 different isoforms [30], [31] and it is difficult to determine the existence of SUMO-conjugated EED forms. Despite this, we were unable to detect any modifications of the slowest migrating EED isoforms suggesting that these isoforms are not SUMO modified *in vivo* (Fig. 1A). Finally, SUMO and UBC9 expression never led to any RbAp48 modifications indicating that RbAp48 does not undergo SUMO conjugation *in vivo* (Fig. 1A). Together, these data suggest that EZH2 and SUZ12 can be targets for SUMO modifications *in vivo*.

To confirm the SUZ12 and EZH2 sumoylation, we tested if the components of the PRC2 complex could be SUMO modified *in vitro*. For this, we reconstituted an *in vitro* SUMO system by purifying from bacteria recombinant SAE1/SAE2 heterodimer (the E1 activating enzyme), UBC9 (the E2 conjugating enzyme) and SUMO-1. As substrate for the reaction we used *in vitro* translated (^35^S-radio-labeled) components of the PRC2 complex. The reaction products were separated by SDS-PAGE and radio-labeled proteins were visualized by autoradiography. As shown in Fig. 1B, SUZ12 can be efficiently attached to SUMO *in vitro*, while EZH2 cannot (Fig. 1B). EED did not show substantial modifications, but a weak modified band could be detected (Fig. 1B). This may suggest that EED can be sumoylated *in vitro* with low efficiency. Finally, RbAp48 did not show any form of SUMO modification in agreement with the *in vivo* data (Fig. 1B).

A possible explanation for the lack of EZH2 sumoylation *in vitro* could be that EZH2 is not folded correctly, when *in vitro* translated, and therefore not recognized efficiently by the sumoylation machinery. Alternatively, other components of the PRC2 complex could be required for EZH2 sumoylation. To test this, we performed the *in vitro* sumoylation reaction using recombinant PRC2 complex purified from insect cells [3] as substrate. As shown in Figure 1C, both SUZ12 and EZH2 were SUMO modified under these reaction conditions, confirming the *in vivo* results presented in Figure 1A. EED was also sumoylated, confirming that EED can be sumoylated *in vitro*. Finally, in agreement with the results presented in Figure 1A and 1B, we did not observe any modifications of RbAp48. Together, these results demonstrate that SUZ12 and EZH2 can be SUMO modified both *in vitro* and *in vivo*, whereas we have been unable to detect sumoylated forms of EED *in vivo* and of RbAp48 *in vitro* and *in vivo*. In addition, SUZ12 always presents a single SUMO modified form (Figure 1A–C), suggesting that it contains a single site of sumoylation. Differently, EZH2 shows multiple bands of modification (Fig. 1C), suggesting that EZH2 can have different sites of SUMO modification and or multiple sumoylations on a single site.

### Identification of SUZ12 SUMO conjugation site

**Figure 2:**
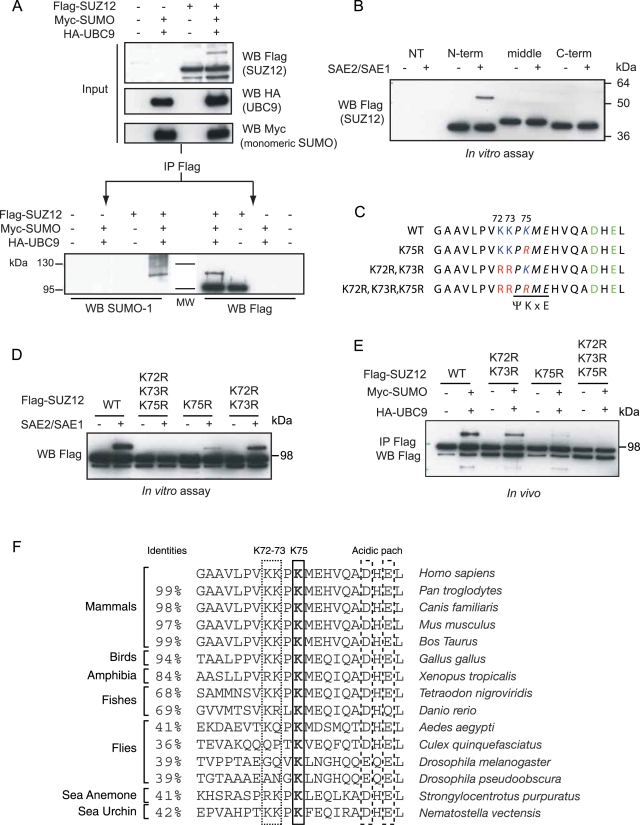
Identification of SUZ12 sumoylation site. (A) Immunopreciptaion (IP) Western blots using antibodies against SUMO-1 and the Flag epitope of 293T cells expressing Flag-SUZ12, SUMO-1 and UBC9, showing that the slower migrating form of SUZ12 corresponds to SUMO-SUZ12. (B) *In vitro* sumoylation assay of Flag immobilised N-terminal (aa 1–283), middle (aa 250–550) and C-terminal (aa 500–739) SUZ12 fragments purified from 293T cells, mapping the SUZ12 SUMO modification site in the first 283 aa. NT-non transfected. (C) Schematic representation of the putative SUMO site predicted at lysine 75 and the three different K to R substitutions to inactivate the potential target lysine. The predicted consensus site is highlighted in italics, the putative target residues are indicated in blue, the acidic patch is indicated in green and the different arginine substitutions are indicated in red. (D) *In vitro* sumoylation assay of the different Flag immobilised SUZ12 mutants shown in panel C, showing that K75 is the preferential site of sumoylation. (E) *In vivo* sumoylation as in panel A of the different SUZ12 mutants shown in panel C, expressed as full-length SUZ12 proteins, demonstrating that K75 is the preferential SUMO site *in vivo*. (F) Alignment of the sequences surrounding K75 between different SUZ12 orthologues. The K75 residue is shown in bold. The full-length SUZ12 homology is indicated on the right as percentage (%) of identities.

Since SUZ12 appears to have one single site of SUMO modification, we chose to focus on characterizing this. To test if the slower migrating band of SUZ12 corresponds to a SUMO-conjugated form, we expressed Flag-tagged SUZ12 in 293T cells together with SUMO-1 and UBC9. Subsequently, we immunoprecipitated SUZ12 from these lysates using an antibody specific for the Flag tag epitope and analyzed the precipitates by western blotting using SUMO-1 and Flag specific antibodies. As shown in Figure 2A, the slower migrating form appearing when SUMO-1 and UBC9 are expressed, corresponds to SUZ12 conjugated to SUMO.

The prediction of potential SUMO sites in the SUZ12 protein identifies more than 10 different potential SUMO consensus motifs. To identify the sites of SUMO modification experimentally, we expressed SUMO-1 and UBC9 together with three different SUZ12 fragments: a N-terminal fragment (amino acids, aa 1–283), a middle fragment (aa 250–550) and a C-terminal fragment (aa 500–739). The different fragments were expressed in 293T cells, immunoprecipitated with a Flag specific antibody and used as substrate for the *in vitro* sumoylation reaction while immobilized on Flag-beads (Fig. 2B). This experiment demonstrates that only the N-terminal fragment of SUZ12 is SUMO- modified *in vitro*, and suggests that the site of sumoylation is within the first 283 amino acids (Fig. 2B). This fragment still contains 6 of the predicted SUMO consensus sites, but only the site predicted at lysine (K) 75 contains an upstream acidic patch consisting of an aspartic and a glutamic acid residue (Fig. 2C). This cluster of acidic residues has been shown to function as an extended SUMO recognition motif that enhances the specificity of SUMO conjugation [28]. Interestingly, two additional lysine residues (K72 and K73) are found adjacent to the K75 (Fig. 2C). In order to test if K75 is the site of SUZ12 SUMO modification, we substituted K72, K73 and K75 with arginines (R) by site-directed mutagenesis and tested the ability of the single K75R, the double K72R, K73R and the triple K72R, K73R, K75R mutants to be SUMO modified. As shown in Fig. 2D, mutation of K75 strongly reduces the sumoylation of SUZ12, whereas mutation of K72 and K73 only lead to a slight reduction in sumoylation. This suggests that K75 is the preferential site of modification, however that also K72 and K73 can function as alternative sites for SUZ12 sumoylation. Consistent with this, mutations of all three lysines completely abrogate the sumoylation of SUZ12.

To understand if the same lysines are used as *in vivo* sumoylation sites, we expressed the different SUZ12 mutants as full-length proteins together with SUMO-1 and UBC9 in 293T cells. As shown in Fig. 2E these experiments showed that the 3 lysines are used as sites of sumoylation *in vivo* and confirmed that K75 is the major site of SUMO modification, also *in vivo*. Importantly, the alignment of the amino acid sequences between SUZ12 orthologues from different organisms revealed that K75 and the residues that form the acidic patch are highly conserved throughout evolution (Fig. 2F). In order to exclude any possible SUZ12 sumoylation in the following experiments we decided to use the triple K72R, K73R, K75R mutations and we refer to this mutant as SUZ12 3KR.

### SUZ12 interacts with UBC9

**Figure 3:**
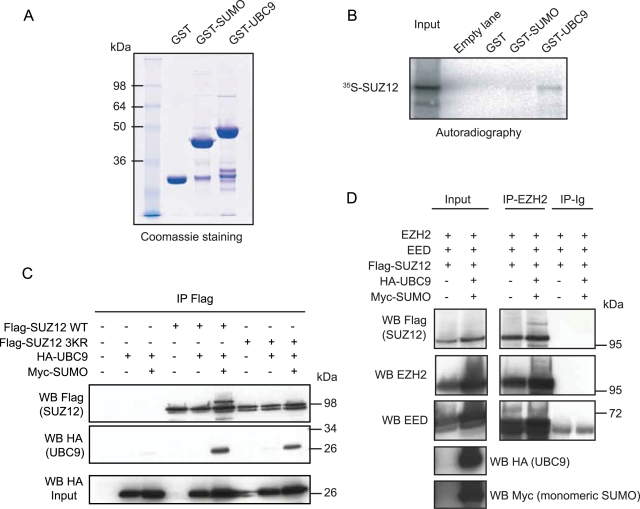
SUZ12 interacts with UBC9 *in vitro* and *in vivo.* (A) Input of recombinant GST-tagged proteins purified from bacteria. (B) *In vitro* binding of *in vitro* translated SUZ12 with GST, GST-SUMO and GST-UBC9, showing that SUZ12 can bind to UBC9. (C) Western blot analysis using the indicated antibodies of SUZ12 immunoprecipitations (IPs) from protein extracts of 293T cells transfected with the indicated constructs showing *in vivo* binding between SUZ12 and UBC9. (D) Western blot analysis using the indicated antibodies of EZH2 IPs from protein extracts of 293T cells transfected with the indicated constructs showing co-immunoprecipitation of the SUZ12 sumoylated form. Mouse Ig served as negative control.

Differently from the ubiquitylation pathway, the SUMO conjugating enzyme UBC9 can interact directly with its substrate and, at least *in vitro*, the activity of E3 ligases is limited to enhancing the efficiency of the sumoylation reaction [32], [33]. Therefore, we tested if UBC9 can bind directly to SUZ12. For this, we performed an *in vitro* binding assay using ^35^S-radio-labeled *in vitro* translated SUZ12 together with recombinant GST-UBC9 or GST-SUMO purified from bacteria (Fig. 3A). As shown in Fig. 3B, UBC9, but not SUMO, can bind to SUZ12 *in vitro*.

To test if this interaction can occur *in vivo*, we co-expressed SUZ12 together with UBC9 alone or in combination with SUMO and UBC9. To exclude that K75 (and K72, K73) is involved in the interaction between SUZ12 and UBC9, we also included the SUZ12 3KR mutant in this experiment. As demonstrated in Fig. 3C, UBC9 can bind efficiently to SUZ12, but only when it is co-expressed with SUMO. Moreover, this interaction is not affected by the 3KR mutation (Fig. 3C). These results confirm that SUZ12 can interact with UBC9 *in vivo* and that the K72, K73 and K75 are not required for this interaction. Furthermore, the fact that efficient UBC9 binding was only observed when co-expressed with SUMO, suggests that loading of SUMO to UBC9 leads to a switch of UBC9 binding affinity for SUZ12.

To better characterize the SUMO modification of SUZ12, we tested if SUMO-SUZ12 can be incorporated into the PRC2 complex. The fact that SUZ12 can be sumoylated *in vitro* when assembled into the recombinant PRC2 complex (Fig. 1C) supports this possibility. To address this *in vivo*, we expressed the three PRC2 members EED, EZH2 and SUZ12 together with SUMO and UBC9 in 293T cells. The result shows that the sumoylated form of SUZ12 could be co-immunoprecipitated by an antibody specific to EZH2 demonstrating that SUZ12 sumoylation does not exclude the protein from the PRC2 complex (Fig. 3D).

### PIASXβ is an E3 SUMO ligase for SUZ12

**Figure 4:**
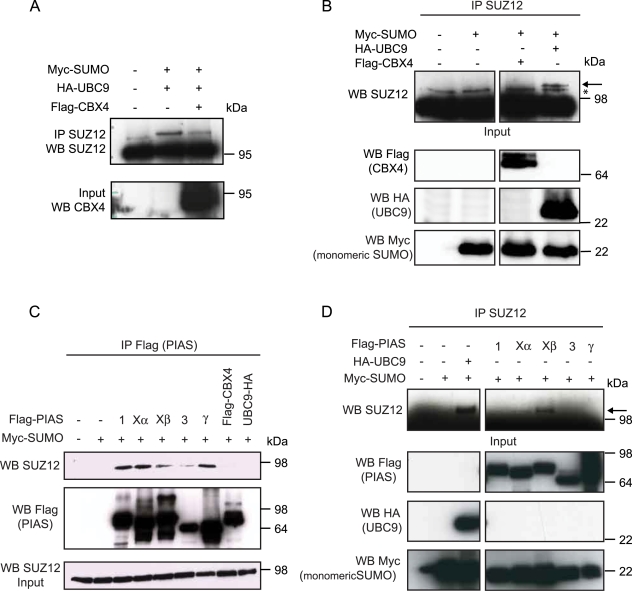
PIASXβ is a potential SUMO E3 ligase for SUZ12. (A) Western blot analysis of 293T cells transfected with SUMO and UBC9 with or without CBX4 expression. (B) Western blot analysis of 293T cells transfected with SUMO with or without CBX4 expression. The SUZ12 SUMO modified form (arrow) and unspecific bands (*) are indicated. (C) Western blot analysis of immunoprecipitations with a Flag-tag specific antibody from 293T cell extracts expressing the indicated Flag tagged proteins showing that endogenous SUZ12 interacts specifically with the members of the PIAS protein family. (D) IP-western blots for endogenous SUZ12 from 293T cells expressing SUMO together with the indicated protein showing that specific expression of PIASXß enhances endogenous SUZ12 sumoylation (arrow).

*In vitro* sumoylation does not require the presence of E3 ligases, but in a physiological context these proteins play an essential role in regulating post-translational sumoylation of proteins [34], [35]. Few proteins have been identified to function as SUMO E3 ligases and these include the members of the PIAS protein family and the PcG protein CBX4. CBX4 is a subunit of the PRC1 complex and since the PRC1 and PRC2 complexes share common target genes and regulatory pathways and since interactions between components of the two complexes have been observed [36], we speculated that CBX4 could function as E3 ligase for SUZ12. For this, we tested if overexpression of CBX4 could enhance SUZ12 sumoylation *in vivo*. We performed this experiment either upon expression of both SUMO and UBC9 (Fig. 4A) or upon expression of SUMO alone (Fig. 4B). In both cases CBX4 overexpression did not enhance SUZ12 sumoylation suggesting that CBX4 does not function as a SUMO E3 ligase for SUZ12. Consistent with this, we did not detect any interactions between SUZ12 and CBX4 (Fig. 4C).

Next, we focused our attention on the PIAS protein family. PIAS are proteins with RING-finger-like domains that have been associated with the sumoylation of a number of different targets. First, we tested if any of the five different PIAS proteins (PIAS 1, Xα, Xβ, 3 and PIASγ) could interact with endogenous SUZ12. Immunoprecipitations of the different PIAS proteins expressed in 293T cells showed that SUZ12 can interact with all members of the PIAS family (Fig. 4C). This interaction is specific since CBX4 expression did not co-precipitate SUZ12 (Fig. 4C). To test if any of these proteins could enhance endogenous SUZ12 sumoylation, we co-expressed the five different PIAS proteins together with SUMO in 293T cells and assayed the levels of SUZ12 sumoylation (Fig. 4D). This experiment demonstrates that PIASXβ expression can specifically enhance the sumoylation of endogenous SUZ12 (Fig. 4D). Together these results suggest that all PIAS proteins have binding affinity for SUZ12, but that only PIASXß can enhance endogenous SUZ12 sumoylation in these conditions.

### SUZ12 3KR mutant does not affect localization and PRC2 activity

**Figure 5:**
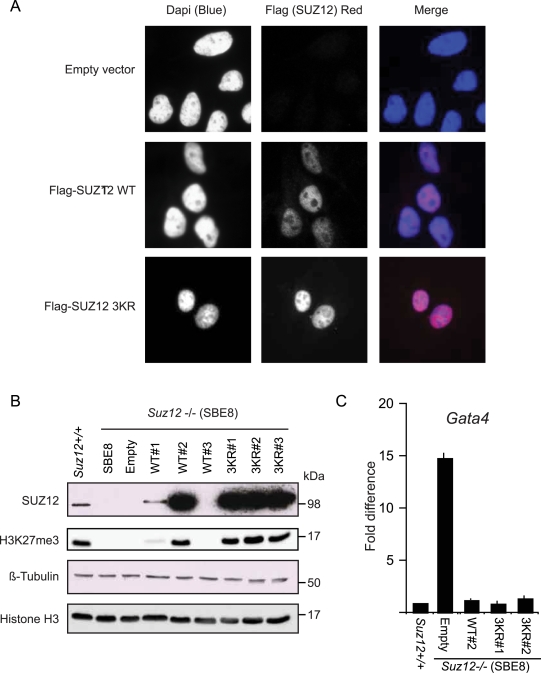
SUZ12 K75 sumoylation is not required for PRC2 activity. (A) Immunofluorecence with a Flag antibody of U2OS cells stably expressing WT or 3KR SUZ12 mutant. (B) Western blot analysis for SUZ12 and H3K27me3 of different Suz12−/− mouse ES cell clones (SBE8) stably expressing either WT or 3KR SUZ12. Suz12+/+ ES cells (E14) served as positive control. Western blots for ß-Tubulin and Histone H3 served as loading control. (C) Expression analysis of *Gata4* in the different clones presented in B by real time quantitative PCR. Relative transcription is normalized to *GAPDH* expression.

To identify a physiological function for SUZ12 sumoylation, we first tested if this modification could affect SUZ12 cellular localization. For this, we stably expressed SUZ12 WT or 3KR mutant in U2OS cells and showed by immunofluorecence staining that mutation of SUZ12 sumoylation site did not affect SUZ12 nuclear localization (Fig. 5A). Secondly, we tested if SUZ12 sumoylation is required for the enzymatic activity of the PRC2 complex. For this, we stably expressed SUZ12 WT or 3KR mutant in mouse *Suz12* −/− Embryonic Stem (ES) cells [37] and analyzed H3K27me3 levels by western blotting (Fig. 5B). As shown in Figure 5B, re-expression of both WT and 3KR mutant SUZ12 restores physiological H3K27me3 levels suggesting that SUZ12 sumoylation is not required for PRC2 enzymatic activity *in vivo*. Finally, we tested if SUZ12 sumoylation could be important for the repressive activity of PRC2. For this we tested if the re-introduction of the 3KR mutant, like wild type SUZ12, could lead to the repression of the PRC2 target gene, *Gata4* [6]. Expression analyses by real-time quantitative PCR show that re-expression of SUZ12 WT and 3KR mutant lead to comparable repression of *Gata4* expression (Fig. 5C). Taken together these data suggest that, under the tested conditions, SUZ12 sumoylation is not required for SUZ12 localization and the catalytic activity of PRC2.

## Discussion

The role of Polycomb group proteins in controlling the expression of a wide range of genes has been extensively studied. These studies have proposed that PcG activity is required for the establishment of specific transcription programs that control cell fate decisions during development [6]–[8], [37]. Consistent with this, loss of PcG function results in embryonic lethality during the gastrulation stage [2], [3], [5], [38]. Although much is now known about the processes controlled by Polycomb complexes, the molecular mechanisms that regulate their recruitment and activity are still not well understood. One possibility could be that post-translational modifications of different PcG proteins may be involved in regulating their activity. For example, phosphorylation of EZH2 mediates its displacement from chromatin and fluctuation of BMI1 phosphorylations during the cell cycle correlates with BMI1 chromatin association [22], [39]. The identification of novel post-translational modifications of PcG proteins is an important challenge that may help to better understand the mechanisms that regulate PcG activity.

In this study we demonstrate that two members of the PRC2 complex, SUZ12 and EZH2, can be sumoylated *in vitro* and *in vivo*. We have shown that SUZ12 contains one single modification site, that EZH2 contains multiple sites of modification and that EZH2 *in vitro* modification requires the assembly of the PRC2 complex. Furthermore, we have mapped the SUZ12 sumoylation site to lysine 75, shown that SUZ12 can interact with UBC9 and identified PIASXß as a potential SUMO E3 ligase for SUZ12.

We have not been able to identify a physiological function for the SUMO modification of SUZ12. Our results suggest that SUZ12 sumoylation is not essential for PRC2 activity in ES cells, but the general low abundance of SUMO modified proteins together with the difficulties in identifying processes where SUZ12 and EZH2 undergo physiological sumoylation make this question extremely difficult to address. Our results indicate that SUZ12 sumoylation is not essential for PRC2 activity in ES cells, however the sumoylation of SUZ12 could be required for specific processes and or in other cellular contexts. Moreover, previous data have shown that protein sumoylation can be transient and that this can be required to mediate specific protein-protein interactions [25]–[27]. A possibility could be that sumoylation of PRC2 is required to provide specific binding affinities. Furthermore, it is possible that sumoylation of the entire PRC2 complex may be required for its activity and that the SUZ12 mutation alone may not be sufficient to lead to a clear phenotype.

In this work we have also identified PIASXß as a potential E3 ligase for SUZ12 suggesting that PIASXß could function as a regulator of PcG activity. The two splice variants of PIASX (Xα and Xβ) are mainly expressed in adult testis in the Sertoli cells, in the germ cells and in the spermatocytes [40], [41]. It is possible that SUZ12 sumoylation may have a role in this specific process and it would be interesting to analyze the PRC2 activity at target genes during spermatogenesis in the absence of functional PIASX proteins. Furthermore, it would also be interesting to analyze the role of SUZ12 sumoylation during spermatogenesis, as well as in other developmental processes, by creating mouse models harboring the SUZ12 3KR mutation.

The fact that K75 is conserved in SUZ12 orthologues with low overall identity (Fig. 2F) suggests that SUZ12 sumoylation could be a conserved feature during evolution. Moreover, the Glutamic (E) and Aspartic acid (D) residues forming the acidic patch C-terminal to the SUZ12 sumoylation site are also highly conserved and, when lost, are substituted with an equivalent acidic residue (E to D substitution in *Drosophila melanogaster* and in *Drosophila pseudoobscura*, Fig. 2F). This could suggest that the acidic patch plays an important role in the sumoylation of SUZ12.

SUZ12 expression is regulated by the pRB/E2F pathway and is required for cellular proliferation of normal and cancer cell lines [3], [18]. Consistent with the role of PcG proteins in cancer formation, the *SUZ12* gene locus is translocated in human Endometrial Stromal Sarcomas (ESS) with high frequency leading to the expression of an uncharacterized fusion protein [42]. Recently it was further proposed that the selection for the expression of the SUZ12 fusion protein in ESS occurs during the benign to malignant transition of these tumors [43]. It is interesting to note that the part of SUZ12 that is involved in this translocation exclude K75 suggesting that the physiological functions of SUZ12 sumoylation will be lost in cancers harboring this translocation. This does not link SUMO directly with the oncogenic effect of this translocation, but highlights that, if SUMO has a role in regulating PRC2 activity, this will be lost in the development of ESS expressing this translocation. Therefore the understanding of the biological role of SUZ12 sumoylation could also contribute to the understanding of the role of this translocation in ESS.

It was reported that a small proportion of the PRC2 complex is present in the cytoplasm of different cell types [44]. In this work it was shown that cytosolic PRC2 regulates Actin polymerization through its ability to bind the guanine nucleotide exchange factor VAV1 and that this activity is linked to Ezh2 requirement for T-cell development *in vivo* [44]. An additional possibility could be that PRC2 sumoylation may play a role in regulating non-chromatin associated PRC2 functions. The fact that we detect sumoylation of nuclear SUZ12 upon SUMO and UBC9 overexpression (data not shown) does not support this but further experiments in this direction will be required to validate such hypothesis.

In conclusion, we have identified a novel potential post-translational modification of components of the PRC2 complex, which may lead to a better understanding of the mechanisms of PcG-mediated regulation of transcription during processes such as proliferation, development, cell fate decision and tumorigenesis.

## Materials and Methods

### Plasmids and antibodies

All constructs encoding the PRC2 complex components have been described previously [3]. All constructs for SUMO, UBC9, SAE1/SAE2 expression were described [45]. Expression constructs for PIAS proteins were described [46]. SUZ12 3KR mutations were obtained by site-directed mutagenesis using the Quick-Change Site Directed Mutagenesis kit (Stratagene). SUZ12 WT and 3KR ORFs were inserted into the HA/Flag EF1-ires-PURO lentiviral expression construct [47] by BamHI subcloning from the pCMV expression constructs described above.

Immunoblottings were performed with the following antibodies: rabbit anti-Suz12 (Upstate), mouse anti-SUZ12 BC23 [48], rabbit anti-β-tubulin (Santa Cruz), mouse anti-EZH2 BD43 [3], mouse anti-EZH2 AC22 [3], mouse anti-EED AA19 [18], rabbit anti-RbAp48 13D10 (Upstate), rabbit anti-HA (Babco), rabbit anti-Flag (Sigma), rabbit anti-Myc (Santa-Cruz), mouse anti-SUMO-1 (Zymed), mouse anti-Cbx4 [9], rabbit anti-H3K27me3 (Upstate), rabbit anti-histone H3 (Abcam), mouse anti-SUZ12 BC23 was generated using recombinant GST-SUZ12 as antigen as described previously [9]. Immunoprecipitations were performed with the following antibodies: mouse anti-Flag M2 (Sigma), mouse anti-EZH2 AC22 [18], mouse anti-SUZ12 2AO9 [48].

### Cell culture and lentiviral production

293T and U2OS cells were cultured in DMEM (Gibco) supplemented with 10% FCS (Hyclone), Pen/Step (Gibco), Glutamax (Gibco). Mouse ES cells were cultured as described [37]. Lentiviral production was carried out as described [49].

### Western blotting and immunoprecipitations

For Western blotting cells were lysed in high salt buffer S300P (50mM Tris-HCl, 300mM NaCl, 0.5% Igepal, 1mM EDTA, 1mM DTT, 1mM PMSF, 1 μg/μl leupeptin, 1 μg/μl aprotinin). For immunoprecipitations, protein G-agarose beads (Zymed) were pre-coupled O/N with the indicated antibodies. Alternatively, mouse anti-Flag M2 beads (Sigma) were used when indicated. Equal amounts of protein lysates (S300P buffer) were used for each immunoprecipitation. Immunoprecipitates were eluted from beads and analyzed by western blotting with the indicated antibodies.

### Expression of recombinant proteins and *in vitro* sumoylation assay

GST-UBC9, GST-SUMO-1 and heterodimeric GST-SAE1-SAE2 fusion proteins were expressed in *E. coli* BL21 for 4 hours at 37°C by addition of 0.2 mM IPTG (Sigma). Recombinant proteins were purified on glutathione-Sepharose beads (Amersham). SUZ12, RbAp48, EED and EZH2 *in vitro* translation was performed with TNT® coupled reticulocyte lysate system (Promega) in the presence of [^35^S]Methionine (Promix, Amersham). The recombinant PRC2 complex expressed in insect cells was prepared as described [3].

The sumoylation reaction was carried out as follows in a total volume of 20 μl at 30°C for 2 hours: 5×reaction buffer (500 mM Tris pH 7.5, 50 mM MgCl_2_, 20 mM ATP, ddH_2_O) 2–5 μl ^35^S-labeled *in vitro* translated target protein or 0.1 μg of recombinant PRC2 complex, 1 μg UBC9, 2 μg SUMO-1 and 0.4 μg SAE1-SAE2 recombinant proteins. The reaction was analyzed by autoradiography for radiolabeled substrates or by western blotting.

### *In vivo* sumoylation

293T cells were transfected with 5 μg of SUMO-1 and 5 μg of UBC9 and harvested after 48 hours. Cells were lysed in denaturing SDS buffer (2% SDS, 100 mM Tris-HCl pH 7.5, 7.5% Glycerol, 40 mM NaCl, 0.4% Igepal, 0.4% deoxycolate, 1mM EDTA, 1mM PMSF, 1 μg/μl leupeptin, 1 μg/μl aprotinin, 15 mM N-ethylmaleimide (NEM)). Immunoprecipitations from samples lysed in denaturing SDS buffer were carried out by diluting the samples 15 times in E1A buffer (50 mM Hepes pH 7.0, 250 mM NaCl, 0.1% Igepal, 5 mM EDTA, 1mM PMSF, 1 μg/μl leupeptin, 1 μg/μl aprotinin) prior to addition of the indicated antibodies.

### GST *in vitro* binding assay

5 μl of [^35^S]Methionine-labeled *in vitro* translated SUZ12 was incubated with 10 μg of GST, GST-SUMO or GST-UBC9 in 1 ml of RV buffer (50 mM Hepes pH 7.5, 150 mM NaCl, 1 mM EDTA, 2.5 mM EGTA, 0.1% Tween-20, 1 mM PMSF, 1 μg/μl leupeptin, 1 μg/μl aprotinin) for 16 hours at 4°C. Samples were centrifuged at 20.000 g for 15 minutes to remove precipitates and incubated with glutathione-Sepharose beads (Amersham) for 2 hours, washed and the immunoprecipitates analyzed by autoradiography.

### Immunofluorescence

U2OS cells were fixed for 10 minutes in Lillie's fixation solution (Merck), permeabilized 5 minutes in 0,1% TritonX-PBS, incubated with the indicated antibodies in 10% FBS for 1 hour, incubated with Alexa Fluor 594 (Invitrogen) secondary antibody for 30 minutes and counterstained with DAPI.

### Quantitative PCR and primers

cDNA preparation and real-time quantitative PCR were performed following manufacturer's instructions (Applied Biosystems). The analysis was performed as described [18]. The primer sequences used for the real-time quantitative PCR were described previously [37].
